# Supplementary material for: Rational Design of Photo-Electrochemical Hybrid Devices Based on Graphene and Chlamydomonas reinhardtii Light-Harvesting Proteins
Source: Sci Rep. 2020 Feb 25;10:3376. doi: 10.1038/s41598-020-60408-5 (PMC7042359; doi:10.1038/s41598-020-60408-5)
Supplement: Supplementary file 1 — Supplementary information [file 41598_2020_60408_MOESM1_ESM.docx]

**RATIONAL DESIGN OF PHOTO-ELECTROCHEMICAL HYBRID DEVICES BASED ON GRAPHENE AND *Chlamydomonas reinhardtii* LIGHT-HARVESTING PROTEINS**

Martha Ortiz-Torres, Miguel Fernández-Niño, Juan C Cruz, Andrea Capasso, Fabio Matteocci, Edgar J. Patiño, Yenny Hernández, A. F. González Barrios.

**SUPPLEMENTARY INFORMATION**

**Supplementary Table S1.** DNA sequences for designed cassettes used in this study.

|  | **DNA sequence** | **Length (bp)** |
| --- | --- | --- |
| **Cassette1** | AAG**CTTCTCCTGCTTCTCCTCCTGCTGCTGCTG**ATGGCTTTCGTTCTGGCTAAATCTTCTGCTTTCGGTGTTGCTGCTAAACCGGTTTCTCGTCGTTCTTCTGTTGCTGTTAAAGCTAGTGCTGTTCCGGAAAACGTTAAAGAAGCTCGTGAATGGATCGACGCTTGGAAATCTAAATCTGGTGGTGCTAAACGTGACGCTGCTCTGCCGTCTTGGATGCCGGGTGCTGACCTGCCAGGCTACCTGAACGGTACCCTGCCGGGTGACTTCGGTTTCGACCCGCTGTACCTGGGTCAGGACCCGGTTAAACTGAAATGGTACGCTCAGGCTGAACTGATGAACGCTCGTTTCGCTATGCTGGCTGTTGCTGGTATCCTGGTTCCGGAACTGCTGTCTAACATCGGTTTCTCTTGGCCGGGGGCAGGTGTTGCTTGGTACGACGCTGGTAAATTCGAATACTTCGCTCCGGCTTCTTCTCTGTTCGGTGTTCAGATGCTGCTGTTCGCTTGGGTTGAAATCCGTCGTTACCAGGACTTCGTTAAACCGGGTTCTGCTAACCAGGACCCGATCTTCACCAACAACAAACTGCCGGACGGTAACGAACCGGGTTACCCGGGTGGTATCTTCGACCCGTTCGGTTGGTCTAAAGGTGACATCAAATCTCTGAAACTGAAAGAAATCAAAAACGGTCGTCTGGCTATGCTGGCTTTCGCTGGTTTCATCGGTCAGGCTTACACCACCGGTACCACCCCGCTGAAAAACCTGTCTACCCACCTGGCTGACCCGTGGTCTACCACCGTTTGGCAGAACGACCTGGCT**AAAAAGAAGAAAAAAAAGAAAAAGAAAAAA**GCTGCAG | 856 |
| **Cassette2** | CTGCAGGT**AAAAAGAAGAAAAAAAAGAAAAAGAAAAAA**ATGGCTTTCGTTCTGGCTAAATCTTCTGCTTTCGGTGTTGCTGCTAAACCGGTTTCTCGTCGTTCTTCTGTTGCTGTTAAAGCTAGTGCTGTTCCGGAAAACGTTAAAGAAGCTCGTGAATGGATCGACGCTTGGAAATCTAAATCTGGTGGTGCTAAACGTGACGCTGCTCTGCCGTCTTGGATGCCGGGTGCTGACCTGCCAGGCTACCTGAACGGTACCCTGCCGGGTGACTTCGGTTTCGACCCGCTGTACCTGGGTCAGGACCCGGTTAAACTGAAATGGTACGCTCAGGCTGAACTGATGAACGCTCGTTTCGCTATGCTGGCTGTTGCTGGTATCCTGGTTCCGGAACTGCTGTCTAACATCGGTTTCTCTTGGCCGGGGGCAGGTGTTGCTTGGTACGACGCTGGTAAATTCGAATACTTCGCTCCGGCTTCTTCTCTGTTCGGTGTTCAGATGCTGCTGTTCGCTTGGGTTGAAATCCGTCGTTACCAGGACTTCGTTAAACCGGGTTCTGCTAACCAGGACCCGATCTTCACCAACAACAAACTGCCGGACGGTAACGAACCGGGTTACCCGGGTGGTATCTTCGACCCGTTCGGTTGGTCTAAAGGTGACATCAAATCTCTGAAACTGAAAGAAATCAAAAACGGTCGTCTGGCTATGCTGGCTTTCGCTGGTTTCATCGGTCAGGCTTACACCACCGGTACCACCCCGCTGAAAAACCTGTCTACCCACCTGGCTGACCCGTGGTCTACCACCGTTTGGCAGAACGACCTGGCT**CTTCTCCTGCTTCTCCTCCTGCTGCTGCTG**TAATCTAGA | 863 |

**10xLeu tag**

**10xLys tag**


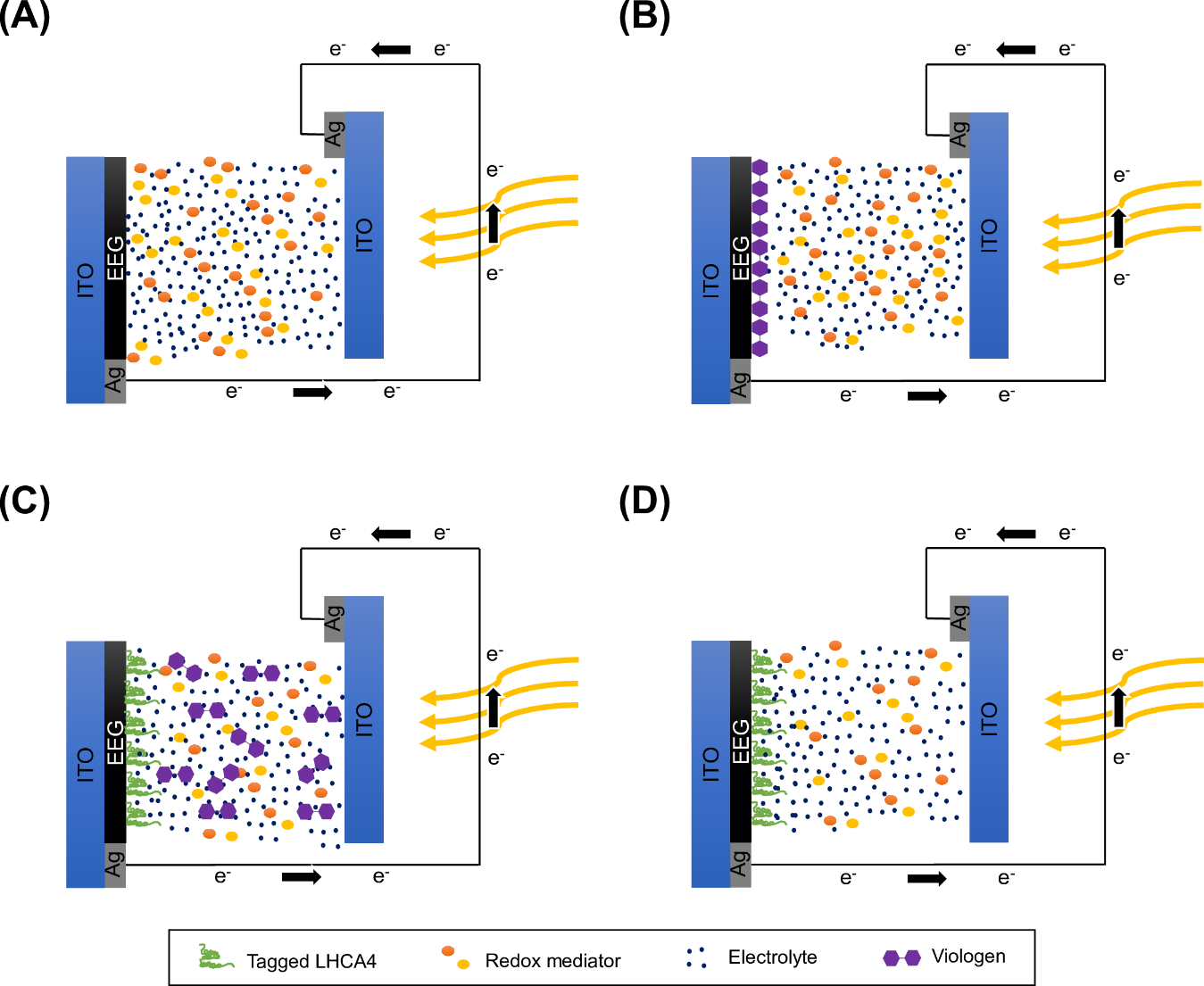


**Supplementary FigureS1**. Schematic representation of tested PEC devices based on different photoanode configurations: (A) without stacked PV and without immobilized proteins as the control device, (B) stacked PV on EEG-based electrode, (C) PV in electrolyte solution (control PEC device assembled with PV-supplemented electrolyte), (D) immobilized LHCA4 proteins (i.e.  C-LHCA4 or N-LHCA4) and (E) immobilized LHC4 proteins on assembled PEC device with supplemented-PV electrolyte.

**
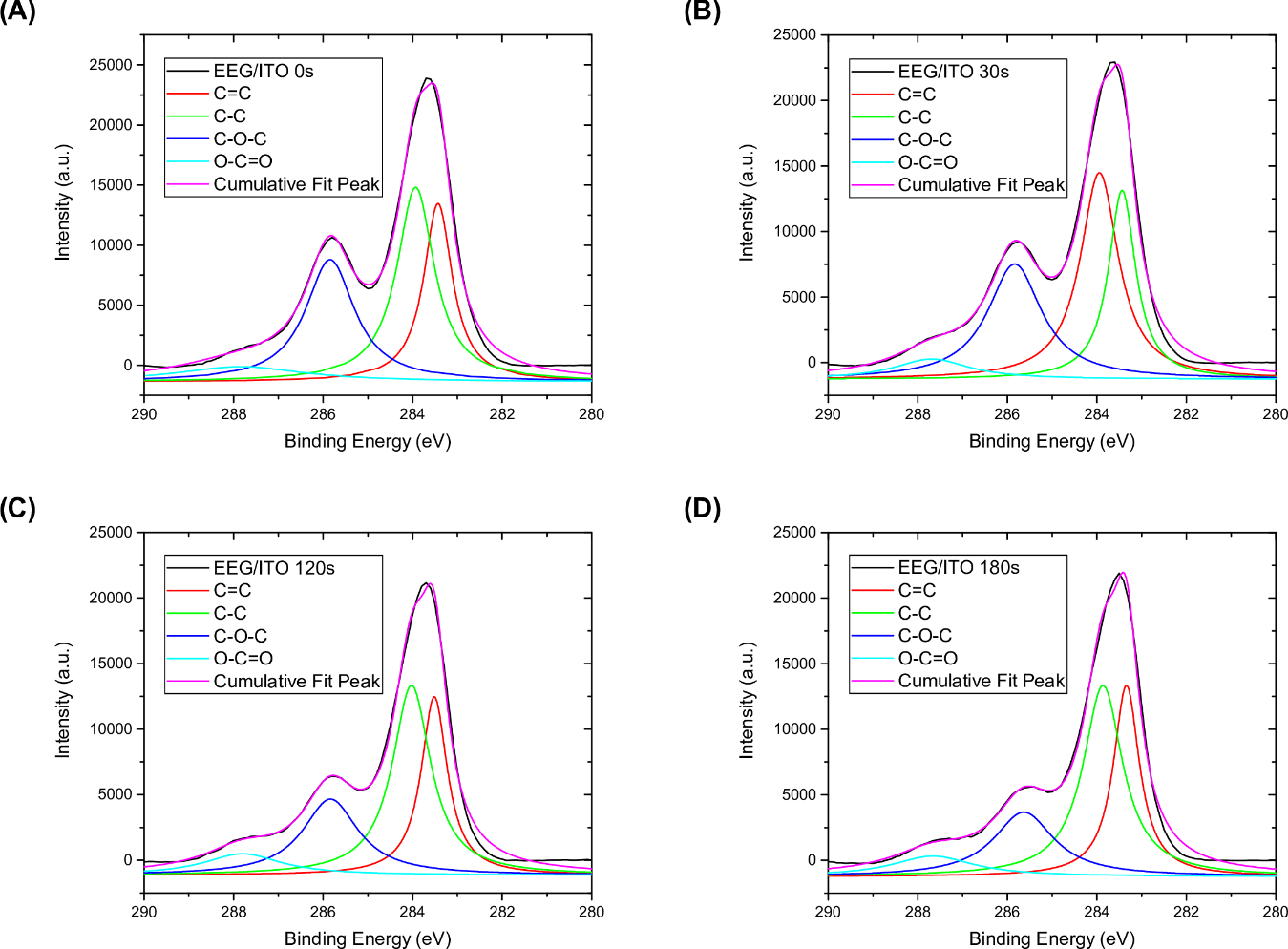
 Supplementary Figure S2.** Binding energies for deconvoluted HR-XPS C1s of EEG-based electrodes under different exposure times (0, 30, 120 and 180 s). Carbon hybridizations sp_3_ and sp_2_ are identified as well as both the carboxylic group (O-C=O) and the ether group (C-O-C).


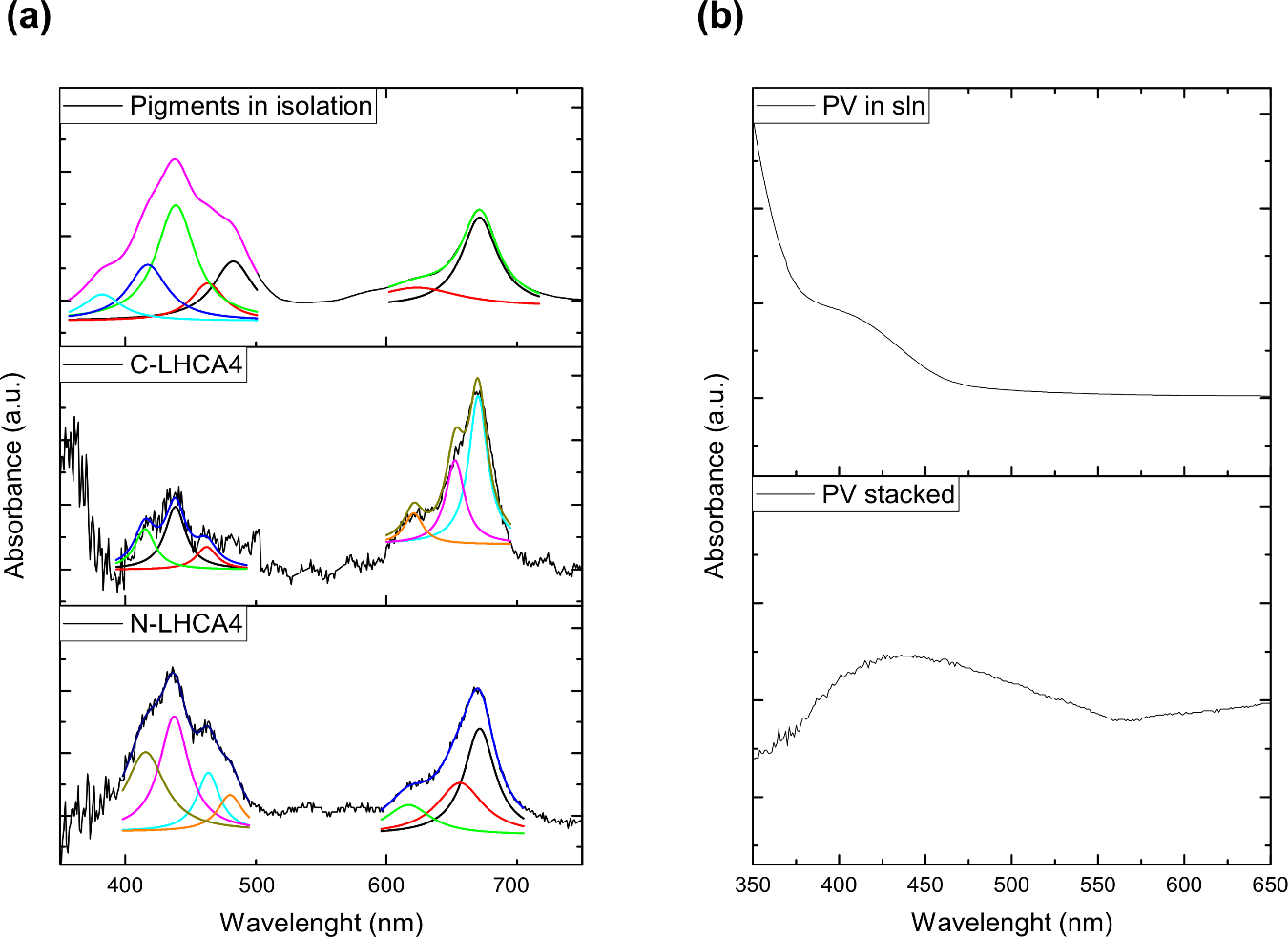


**Supplementary Figure S3.** Absorption spectrophotometry of the photoanodes ensembled in this study. (a) Deconvoluted absorption spectra of isolated pigments in aqueous solution, C-LHCA4 and N-LHCA4 immobilized on the EEG-based electrode with the pigments added before protein immobilization as explained in the Fig. 4, respectively. (b) Absorption spectra of both the PV in solution and the EE-based electrode with PV stacked.


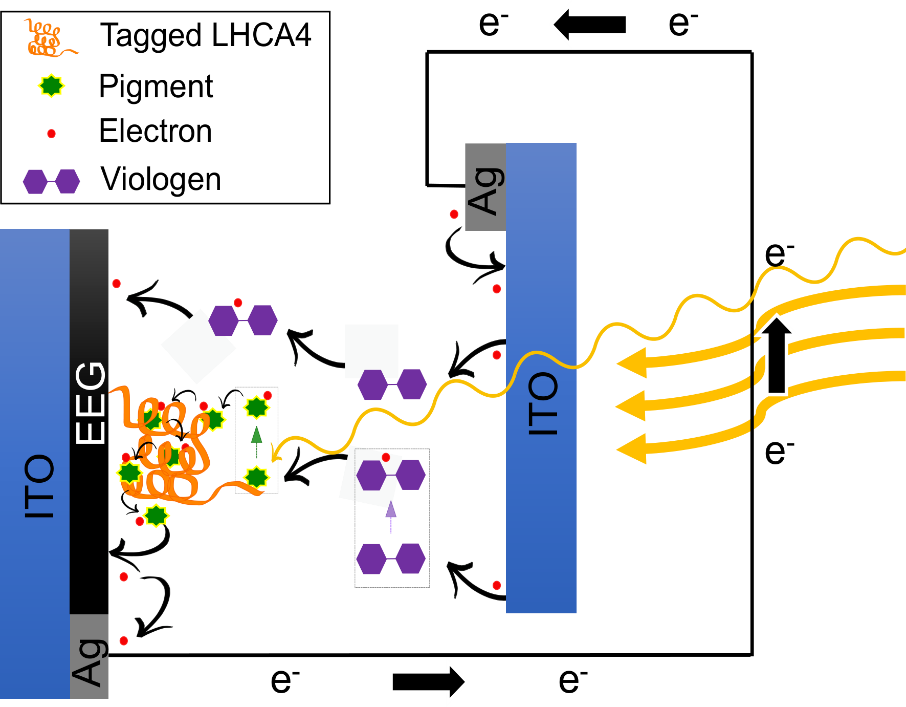


**Supplementary Figure S4.** Schematic of the photoelectrochemical process on the DSSC with LHCA4 and the viologen molecule. The photocurrent might be observed from two likely contributions: the redox reactions of the viologen and the charge transfer through the protein due to the weak interaction within the pigments. This last contribution might be initiated either by the light source or by a charge transfer from the oxidized species of the viologen molecule to the pigment. Additionally, the created holes after the pigment excitation might be recombined by the reduced species of the viologen. Finally, as in any other photoelectrochemical cell, the charge might be with the aid of the KCl present in the electrolyte.





**Supplementary Figure S5.** Photocurrent measurements recorded for EEG-based electrodes with immobilized proteins: (a) C-LHCA4 and (b) N-LHCA4. For both protein-based devices, the displayed photocurrent (at the bottom) was compared with the photocurrent of similar devices without neither C-LHCA4 nor N-LHCA4, respectively (at the top) and, without pigments (in the middle).

**
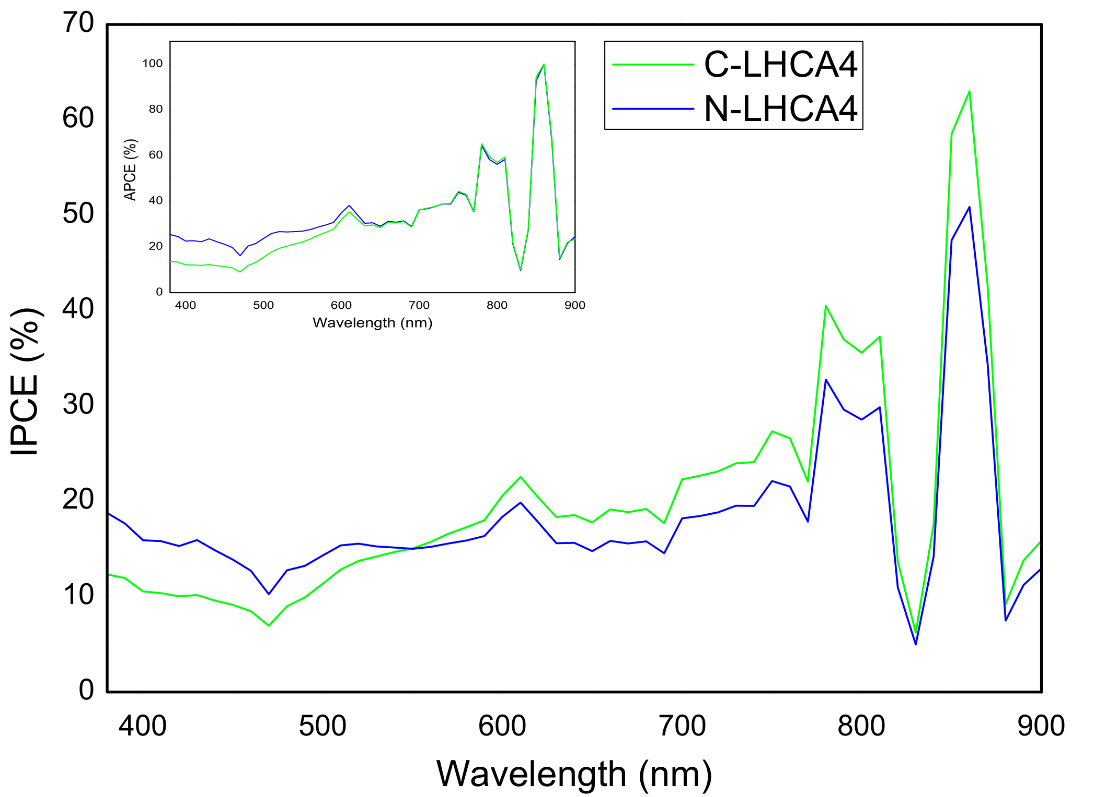
**

**Supplementary Figure S6.** IPCE spectra for the devices C-LHCA4 and N-LHCA4. Both spectra were recorded using a monochromatic light source based on monochromator and a 150W Xenon lamp. A metallic mask was used to illuminate a spot with an area of 0.1 cm^2^. *Inset:* APCE calculated from the IPCE and absorption data.
